# SLEEP REGULARITY, SLEEP TIMING, AND COGNITIVE PERFORMANCE AMONG WOMEN ENTERING EARLY OLDER AGE

**DOI:** 10.1093/geroni/igad104.2032

**Published:** 2023-12-21

**Authors:** Carol Derby, Michelle Hood, Martica Hall, Hadine Joffe, Rebecca Thurston, Howard Kravitz, Leslie Swanson

**Affiliations:** Albert Einstein College of Medicine, Bronx, New York, United States; University of Michigan, Ann Arbor, Michigan, United States; University of Pittsburgh, Pittsburgh, Pennsylvania, United States; Harvard Medical School at Brigham and Women’s Hospital, Boston, Massachusetts, United States; University of Pittsburgh, Pittsburgh, Pennsylvania, United States; Rush University Medical Center, Chicago, Illinois, United States; University of Michigan, Ann Arbor, Michigan, United States

## Abstract

Poor sleep health is a modifiable behavior linked to cognitive impairment risk. While prior work has focused primarily on sleep quantity and quality, emerging evidence suggests the importance of sleep timing and regularity. We examined whether sleep timing and regularity were associated with cognitive performance within a diverse cohort of women ages 60.5-72.2 years, and tested whether associations were independent of sleep duration and quality (wake after sleep onset). The sample included 1178 SWAN women who completed 4-7 nights of actigraphy and cognitive testing at the 15th SWAN follow-up in 2015-2016, [Mean age 65.5 (± 2.63 years); 25% Black, 12% Chinese, 6% Hispanic White, 11% Japanese, 46% Non-Hispanic White]. Sleep timing was defined as the midpoint time between sleep onset and waking, averaged across nights and was dichotomized as healthy (2am – 4am) or unhealthy (outside 2am – 4am). Sleep regularity was defined as the standard deviation of the sleep midpoint across nights. Associations of sleep regularity and timing with verbal episodic memory (East Boston Memory immediate (EBMT-I) and delayed (EBMT-D)), and processing speed (Symbol Digit Modalities) were examined using linear regression. Covariates included sleep duration and quality, age, race/ethnicity, education, economic hardship, depression, comorbidities, medications, alcohol use, vasomotor symptoms and testing language. Greater sleep irregularity was associated with worse verbal memory (EBMT-I β= -0.29; p=0.02; EBMT-D β= -0.36; p=0.007). Sleep midpoint outside the healthy range was associated with slower processing speed (β= -1.48; p=0.017). Sleep timing and regularity may be important targets for interventions aimed at preserving cognitive health.

